# Stability of c-Myc Protein in Early S Phase Is Regulated by the Interaction with PCNA

**DOI:** 10.3390/ijms27062745

**Published:** 2026-03-18

**Authors:** Miriana Cardano, Ornella Cazzalini, Giusy Maraventano, Lucia A. Stivala, Laura Zannini, Ennio Prosperi

**Affiliations:** 1 Istituto di Genetica Molecolare “Luigi Luca Cavalli-Sforza”, Consiglio Nazionale delle Ricerche (CNR), Via Abbiategrasso 207, 27100 Pavia, Italylaura.zannini@igm.cnr.it (L.Z.); 2 Dipartimento di Medicina Molecolare, Università degli Studi di Pavia, Via Ferrata 9, 27100 Pavia, Italy; ornella.cazzalini@unipv.it (O.C.); luciaanna.stivala@unipv.it (L.A.S.)

**Keywords:** c-Myc, PCNA, PCNA-interacting protein box, CUL4A, CRL4 ubiquitin ligase

## Abstract

The transcription factor c-Myc is known to regulate DNA replication via a non-transcriptional mechanism by interacting with proteins of the pre-replicative complex. In addition, c-Myc localizes to DNA replication foci, similarly to Proliferating Cell Nuclear Antigen (PCNA); however, the significance of this localization remains unclear. Here, we investigated whether c-Myc interacts with PCNA and analyzed the possible function of this association. We found a conserved interaction motif, the PCNA-interacting protein (PIP) box, in the N-terminal region of c-Myc. Confocal microscopy analysis showed co-localization with PCNA in early S-phase, but not in late S-phase cells. Co-immunoprecipitation from cell extracts and pull-down of recombinant proteins indicated a direct physical association between c-Myc and PCNA, which was confirmed in situ by the Proximity Ligation Assay (PLA). Further experiments demonstrated that c-Myc interacts with CUL4A and DDB1, components of the Cullin Ring E3 ubiquitin ligase 4 (CRL4) complex, in which PCNA functions as a cofactor. Mutations in the PIP box of c-Myc, as well as depletion of CUL4A by RNA interference, resulted in an increased stability of c-Myc protein. These results suggest that the interaction with PCNA functionally contributes to the regulation of c-Myc stability in early S phase via the CRL4 complex.

## 1. Introduction

The Myc family of transcription factors, including c-Myc, N-Myc, and L-Myc members, are important regulators of cell proliferation and apoptosis and play a fundamental role in stimulating cell growth. The three Myc genes are often amplified in several tumor types, making their overexpression a common feature of many human cancers [1,2,3]. For this reason, the regulation of Myc is an important parameter considered in studies aiming at developing new therapeutic strategies for cancer treatment [4,5].

The primary role of c-Myc is to promote cell cycle progression by driving entry into S-phase, activating cyclins and CDK complexes, and releasing cell cycle brakes [6]. However, additional functions have been described, including direct, non-transcriptional control of DNA replication [7,8], and more recently, involvement in base excision repair [9].

In the context of DNA replication, c-Myc was found to interact with proteins of the pre-replicative complex, including all MCM2–7 subunits, Orc2, Cdc6, and Cdt1 [7]. Overexpression of c-Myc induced DNA replication stress by increasing the replication origin activity [7]. More targeted studies showed that c-Myc can alter the density of replication origins, and its deregulation resulted in elevated fork stalling or collapse, effects that were also observed upon Cdc45 overexpression [10,11]. Interestingly, c-Myc was found to co-localize with BrdU in early S-phase cells [7,10], and c-Myc localization at replication foci appears to overlap with that of Proliferating Cell Nuclear Antigen (PCNA) in early S-phase cells [10]. PCNA is a well-known protein involved in DNA replication and repair, as well as in other functions related to DNA metabolism and in cell processes such as apoptosis [12,13,14,15]. Typically, PCNA localizes in S-phase cells at DNA replication foci, i.e., the sites of ongoing DNA synthesis [16,17,18]. Therefore, the co-localization of c-Myc with BrdU and the foci distribution in S phase, similar to that of PCNA, prompted us to investigate whether the two proteins interact and to understand the function of this association [19].

## 2. Results

### 2.1. c-Myc co-Localizes with PCNA Only in Early S Phase

**Figure 1:**
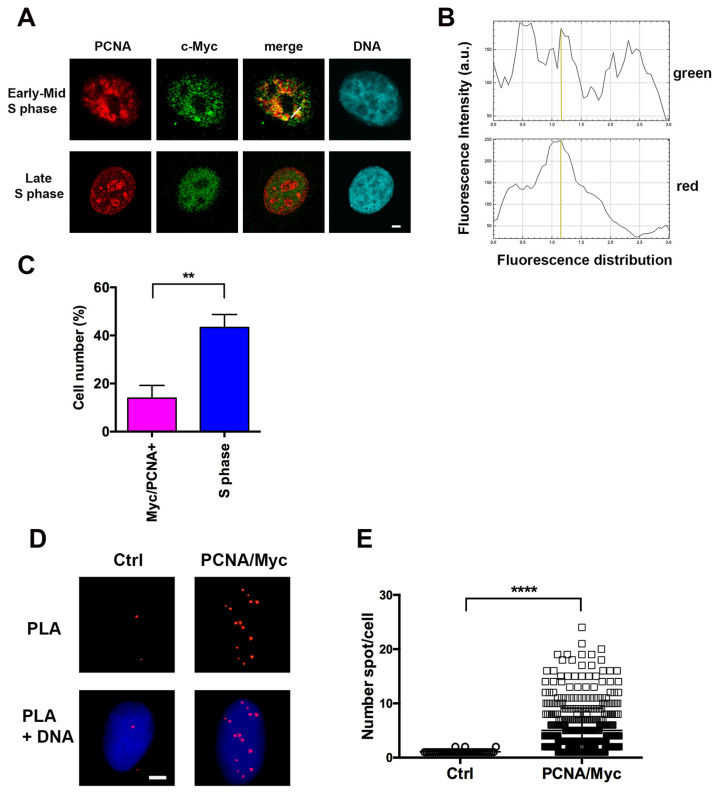
Co-localization and proximity of c-Myc with Proliferating Cell Nuclear Antigen (PCNA) in S-phase cells. (**A**) Confocal microscopy analysis of immunofluorescence signals of chromatin-bound PCNA (red fluorescence) and c-Myc (green fluorescence) proteins in HeLa cells. Scale bar = 2 μm. (**B**) Graphic representation of fluorescence spatial distribution of green and red fluorescence signals along the white line shown in the “merge” image of panel (**A**). A peak of coincidence of both signals is indicated by the yellow line. (**C**) Average counts of cells in S phase and of cells showing co-localization of c-Myc and PCNA. Data are mean values ± standard deviations (s.d.) from 3 independent experiments. ** *p* < 0.01. (**D**) Proximity Ligation Assay (PLA) of c-Myc and PCNA proteins shown by red spots in cells immunostained with anti-PCNA (PC10) and anti-Myc (Y69) antibodies (PCNA/Myc) vs. negative control (Ctrl) stained with a single antibody (PC10). Scale bar = 2 μm. (**E**) Scatter plot representation of the number of PLA spots/cell nucleus in PCNA/Myc vs. negative control samples. Values were collected in 3 different experiments. **** *p* < 0.0001.

Previous studies reported that c-Myc co-localizes with BrdU at sites of DNA replication, but only a comparison with the distribution of PCNA was reported, without direct analysis of their co-localization [10]. To explore this, we initially investigated the proximity of c-Myc and PCNA using confocal fluorescence microscopy following immunofluorescence staining of the proteins. Experiments were performed with in situ hypotonic lysis, a procedure that allows selective detection of chromatin-bound PCNA in S-phase cells [16,17,18], i.e., when c-Myc is present at DNA synthesis sites [7,10]. Interestingly, confocal microscopy analysis showed c-Myc co-localization with PCNA in early, but not in late S-phase cells (Figure 1A). However, the degree of co-localization appeared only partial, as shown by the green and red fluorescence distribution in a selected region of the nucleus (Figure 1B). In addition, quantification of the cells showing co-localization of chromatin-bound PCNA and c-Myc revealed that this positivity was limited to a subset of S-phase cells, occurring in only about 30% of S-phase cells, corresponding to less than 10% of total cells (Figure 1C). To further assess a potential interaction between c-Myc and PCNA, the “Proximity Ligation Assay” (PLA) was performed under the same conditions as the co-localization analysis, i.e., after in situ hypotonic lysis to release proteins not associated with chromatin. As shown in Figure 1D, c-Myc and chromatin-bound PCNA were in close proximity, as indicated by the significantly higher number of PLA signals in samples stained with both antibodies compared with negative controls stained with a single antibody (mean value 5 vs. 1, *p* < 0.0001), as shown in Figure 1E.

### 2.2. c-Myc Interacts with PCNA

**Figure 2:**
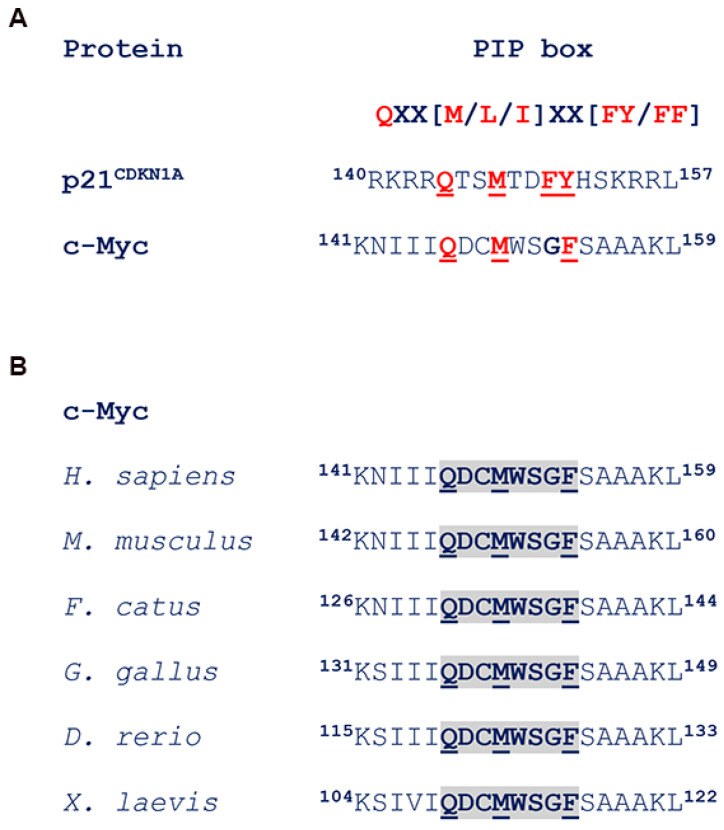
Identification of a PIP box in the c-Myc sequence. (**A**) The canonical PIP box sequence of p21CDKN1A is compared with that present in the c-Myc amino acid sequence in the region encompassing residues 141–159. Conserved residues are shown in red. (**B**) The c-Myc sequence in the PIP box region of the human gene is compared with that in other mammals and in vertebrates. Conserved residues are in bold outline.

Most PCNA partners share a characteristic 8-amino acid stretch called the PCNA-interacting protein (PIP) motif, or PIP box [20]. A well-known example of this sequence is found in p21^CDKN1A^, a PCNA interactor that regulates multiple functions in DNA replication and repair [21,22,23]. Interestingly, a putative PIP box was identified in the human c-Myc sequence at residues 146–153 (Figure 2A). This region is highly conserved among mammals and other vertebrates, including chickens, Xenopus, and zebrafish, as shown in Figure 2B.

**Figure 3:**
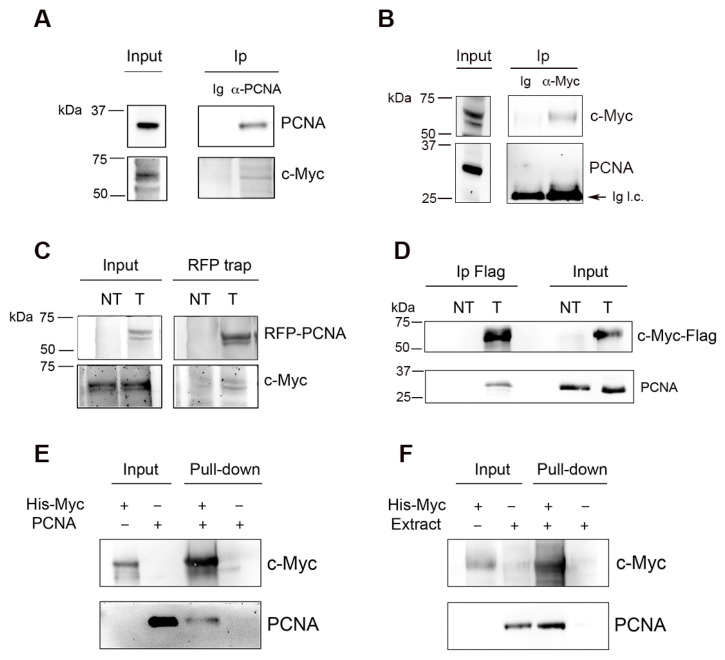
Analysis of the interaction between c-Myc and PCNA. (**A**). Immunoprecipitation (Ip) assay performed with anti-PCNA antibodies (PC10 plus Ab5) in HeLa whole cell extracts. Control Ip was performed with an irrelevant antibody (Ig). An immunoblot was performed with PC10 antibody for PCNA and Y69 for c-Myc. (**B**) Immunoprecipitation (Ip) assay performed with anti-Myc antibody (Y69) in HeLa whole cell extracts. Control Ip was performed with an irrelevant antibody (Ig). The arrow indicates bands of Ig light chains (Ig l.c.). (**C**) Immunoprecipitation of RFP-PCNA and c-Myc was performed with RFP trap (RFP nanobody) in HeLa cells transfected (T) or non-transfected (NT) with the mRFP-PCNAL2 construct (43). Immunoblots were performed with anti-RFP (PCNA) or Y69 antibody (c-Myc). (**D**) Immunoprecipitation by anti-Flag antibody of c-Myc-Flag and PCNA in HeLa cells transfected (T) or non-transfected (NT) with c-Myc-Flag construct. Immunoblots were performed with anti-Flag (Myc-Flag) or PC10 antibody (PCNA). (**E**) Pull-down assay of recombinant PCNA with His-tagged recombinant c-Myc, performed with Ni^+^-NTA beads. Immunoblots were performed with PC10 (PCNA) or Y69 antibody (c-Myc). (**F**) Pull-down assay of endogenous PCNA from HeLa cell extract with His-tagged recombinant c-Myc, performed with Ni^+^-NTA beads. Immunoblots were performed with PC10 (PCNA) or Y69 antibody (c-Myc).

To assess whether c-Myc interacts with PCNA, we performed an immunoprecipitation assay (Ip) on HeLa cell extracts using antibodies against either c-Myc or PCNA. Ip results with anti-PCNA antibodies resulted in co-immunoprecipitation of c-Myc (Figure 3A), whereas Ip with anti-c-Myc antibody did not detect PCNA (Figure 3B). We then examined whether the interaction could be more easily detected upon protein overexpression. To this end, HeLa cells were transfected with a construct expressing RFP-tagged PCNA or Flag-tagged c-Myc protein. The results showed that following the overexpression of either RFP-PCNA or c-Myc-Flag, Ip performed with the respective anti-tag antibodies (RFP or Flag) contained both proteins (Figure 3C,D). Finally, to further prove that this association was not mediated by other proteins, recombinant His-tagged c-Myc was incubated together with untagged recombinant PCNA, and a pull-down assay was performed using Ni^+^-NTA beads. Immunoblot analysis of isolated material revealed that recombinant PCNA was isolated together with His-tagged c-Myc protein (Figure 3E). Pull-down of PCNA was also observed when the recombinant His-tagged c-Myc was incubated with whole HeLa cell extract (Figure 3F).

### 2.3. Interaction of c-Myc with PCNA and Components of the CRL4 Complex

**Figure 4:**
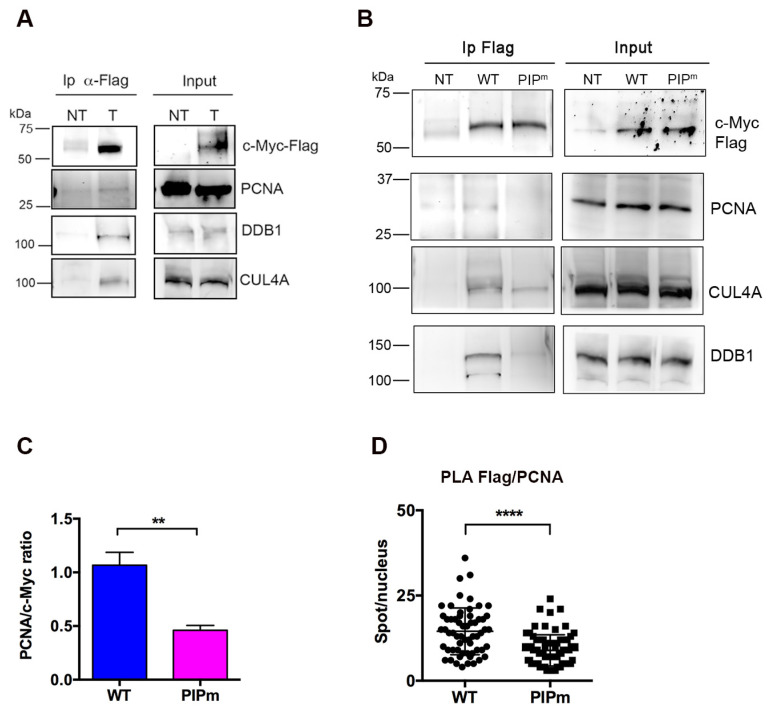
Interaction of c-Myc and PCNA with CRL4 complex components. (**A**). Immunoprecipitation (Ip) assay performed with anti-Flag antibody in HeLa cells transfected (T) or non-transfected (NT) with c-Myc-Flag construct. (**B**) Immunoprecipitation (Ip) assay performed with anti-Flag antibody in HeLa cells transfected (T) or non-transfected (NT) with c-Myc^WT^-Flag or c-Myc^PIPm^-Flag constructs. (**C**) Quantitative analysis of the amount of PCNA co-immunoprecipitated with Flag-tagged c-Myc. The mean values (±s.d.) were obtained by densitometric analysis of PCNA bands normalized to those of c-Myc-Flag immunoprecipitated in three biological replicates (** *p* < 0.01). (**D**) PLA was performed in HeLa cells expressing c-Myc^WT^-Flag or the PIPm construct. The antibody pair used consisted of anti-Flag (mouse) and anti-PCNA (rabbit) antibodies. The results are spot counts in at least 60–70 cells for each construct from two independent biological replicates (**** *p* < 0.0001).

To elucidate the role of c-Myc-PCNA interaction, we investigated whether it could facilitate the recognition of c-Myc by the Cullin Ring E3 ubiquitin ligase 4 (CRL4) complex, which mediates the ubiquitination of relevant substrates for proteasomal degradation using PCNA as a cofactor [24,25,26]. For this purpose, Ip was performed using an anti-Flag antibody on extracts from HeLa cells expressing the c-Myc-Flag, followed by Western blot analysis. To verify the presence of components of the CRL4 complex in the immunoprecipitated material, the analysis was performed with anti-DDB1 and anti-CUL4A antibodies. As shown in Figure 4A, both proteins co-immunoprecipitated c-MycFlag, suggesting that the interaction with PCNA could facilitate c-Myc recognition by the CRL4 complex and contribute to its degradation. To confirm this hypothesis, a mutant form of c-Myc-Flag was generated by replacing key residues in the putative PIP-box with alanine (Q146A, M149A, F153A). Expression of this mutation, named c-Myc^PIPm^-Flag, in HeLa cells confirmed nuclear localization and SDS-PAGE migration similar to the WT protein (Supplementary Figure S1A,B). The ability of the mutant protein c-Myc^PIPm^-Flag to interact with PCNA and with the CRL4 complex was then analyzed by immunoprecipitation with anti-Flag antibody. Western blot analysis of precipitated proteins revealed that the amount of PCNA, and to a lesser extent DDB1 and CUL4A, was reduced in cells expressing the c-Myc^PIPm^-Flag mutant protein compared with cells expressing the c-Myc^WT^-Flag protein (Figure 4B). Quantitative densitometry analysis confirmed that PCNA co-immunoprecipitated with c-Myc at approximately 50% of the level observed with the WT protein (Figure 4C). To further validate these findings, we performed PLA in HeLa cells expressing exogenous c-Myc^WT^-Flag or the mutant PIPm form. For this assay, the anti-Flag mouse antibody was used to detect exogenous c-Myc, together with a rabbit polyclonal antibody against PCNA. As shown in Figure 4D, expression of c-Myc^PIPm^-Flag protein resulted in a significant reduction in the number of PLA spots compared with c-Myc^WT^-Flag protein, confirming that mutations in the PIP box influence the proximity between c-Myc and PCNA.

### 2.4. Interaction with PCNA Regulates c-Myc Degradation

**Figure 5:**
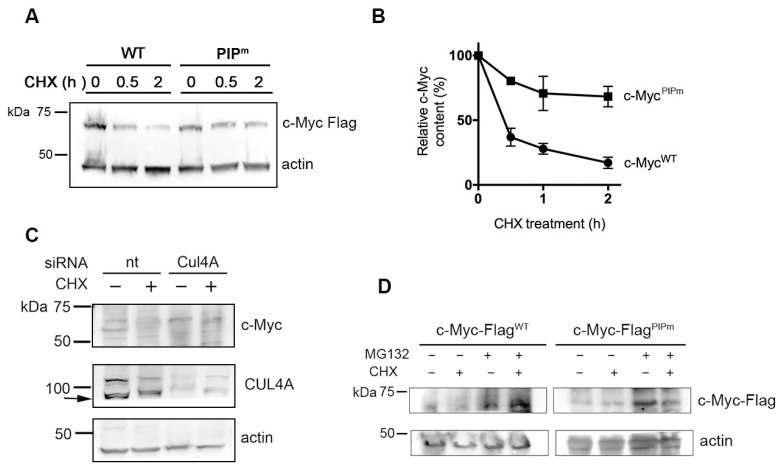
Stability of exogenous c-Myc^WT^-Flag and c-Myc^PIPm^-Flag proteins. (**A**) Western blot analysis of c-Myc-Flag WT and PIPm exogenous proteins in whole HeLa cell extracts after incubation for the indicated periods with CHX. Actin is shown for protein loading control. (**B**) Relative content of exogenous c-Myc-Flag WT or PIPm proteins was obtained by band densitometry and normalized to actin content. Mean values ± s.d. of three independent experiments are shown. (**C**) Western blot analysis of endogenous c-Myc protein in whole HEK cell extracts after incubation for 72 h with non-targeting (nt) or CUL4A-targeting (Cul4A) siRNA. Cells were incubated in the presence (+) or in the absence (−) of CHX during the last two hours of siRNA treatment. The arrow indicates the expected position of CUL4A. (**D**) Western blot analysis of c-Myc-Flag WT and PIPm exogenous proteins in whole HeLa cell extracts after treatment with the proteasome inhibitor MG132, both in the presence and in the absence of CHX. Actin is shown for protein loading control.

To investigate the stability of the mutant vs. the wild-type c-Myc protein, cells were treated with cycloheximide (CHX) for up to 2 h to block de novo protein synthesis. Extracts from untreated and CHX-treated cells were analyzed by Western blot to determine the levels of WT and mutant c-Myc-Flag proteins. The results shown in Figure 5A,B indicate that the c-Myc^PIPm^-Flag protein was degraded with slower kinetics than the corresponding c-Myc^WT^-Flag, suggesting that the interaction with PCNA could facilitate the recognition of c-Myc by the CRL4 E3 ligase complex, in which PCNA participates in substrate recognition [24,25,26]. To further prove this hypothesis and confirm the involvement of the CRL4 complex in the degradation of c-Myc, CUL4A expression was silenced by RNA interference. HEK cells were used for these experiments due to their higher transfection efficiency. Cells were incubated for 72 h with CUL4A-targeting siRNA molecules, followed by incubation with CHX for 2 h to block new protein synthesis. The results shown in Figure 5C indicate that the CUL4A protein level was significantly reduced by specific siRNA treatment compared with non-targeting (nt) siRNA. Control cells treated with non-targeting siRNA showed a decrease in c-Myc levels in the presence of CHX compared with untreated samples. In contrast, the depletion of CUL4A resulted in the resistance of c-Myc to degradation, both in the absence and in the presence of CHX (Figure 5C). Finally, the involvement of the proteasome in the c-Myc-Flag degradation was also analyzed by using the inhibitor MG132 in both the presence and absence of CHX. The results confirmed that c-Myc-Flag degradation occurred through the proteasome, as treatment with MG132 led to the accumulation of both WT and PIPm mutant c-Myc-Flag proteins (Figure 5D).

### 2.5. PCNA Regulates c-Myc Stability After DNA Damage

**Figure 6:**
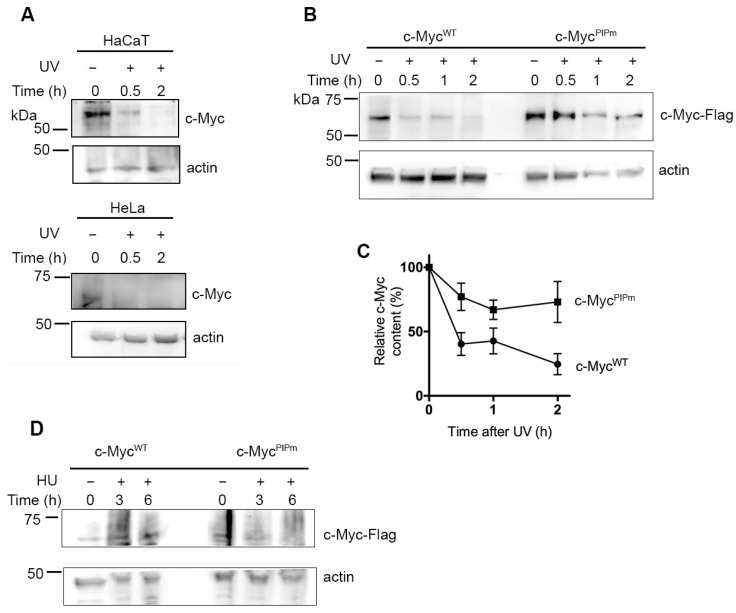
Stability of c-Myc protein after DNA damage. (**A**) Western blot determination of c-Myc protein stability in HaCaT and HeLa cells after UV-induced DNA damage. Actin is shown for protein loading control. (**B**) Western blot determination of exogenous c-Myc^WT^-Flag and c-Myc^PIPm^-Flag protein stability after UV-induced DNA damage. Actin is shown for protein loading control. (**C**) Relative content of exogenous c-Myc-Flag WT and mutant proteins in HeLa cells, normalized to actin content, was obtained by band densitometry. Mean values ± s.d of three independent experiments are shown. (**D**) Western blot determination of exogenous c-Myc^WT^-Flag and c-Myc^PIPm^-Flag protein stability after HU treatment (2.0 mM) for the indicated periods of time. Actin is shown for protein loading control.

Several proteins degraded via the CRL4 complex through the interaction with PCNA are subjected to a similar fate following DNA damage [25,27]. For this reason, we analyzed c-Myc protein levels after DNA damage induced by UV irradiation. Initially, degradation of endogenous c-Myc was analyzed in HaCaT and HeLa cells. c-Myc levels decreased rapidly, with degradation detectable as early as 30 min after irradiation and becoming almost undetectable after 2 h (Figure 6A). Then, we investigated whether CRL4 and PCNA could play a role in c-Myc degradation, also following DNA damage. HeLa cells expressing c-Myc^WT^-Flag or c-Myc^PIPm^-Flag were irradiated with UV light and collected after various time points. Western blot analysis showed that c-Myc^PIPm^-Flag was degraded with slower kinetics compared with the WT protein (Figure 6B,C). To establish whether another type of stress (e.g., the block of DNA replication) affects the c-Myc stability, HeLa cells expressing exogenous c-Myc-Flag WT or PIPm proteins were treated for 3 or 6 h with HU, a well-known inhibitor of DNA replication. Cells were then collected and analyzed by Western blot. The results (Figure 6D) showed that the levels of exogenous c-Myc-Flag^WT^ protein were not significantly altered by the HU treatment. A similar behavior was observed in cells expressing the PIPm protein, although a slight decrease was detected after prolonged treatment. The differences observed between UV and HU treatment may be dependent on the type of damage, and further studies will be required to explain these results.

## 3. Discussion

The presence of c-Myc in S-phase cells was reported several years ago, demonstrating its involvement in the control of DNA replication independently of transcriptional activity [7,8,11]. In this study, we verified that c-Myc localizes in close proximity to PCNA, the master of DNA replication coordinating multiple protein interactions during S phase [12,13,14]. However, since PCNA also participates in DNA repair and in additional processes taking place in the cytoplasm [14,15], the identification of a partnership with c-Myc prompted us to investigate the significance of this connection.

The evidence that co-localization with PCNA is restricted to cells in early, but not late, S phase suggests that the association between the two proteins is temporally limited, consistent with previous reports describing co-localization of c-Myc at early DNA synthesis sites [7,10]. The transient nature of this interaction might explain why PCNA was not identified in previous studies of the Myc interactome [28,29,30]. In fact, we failed to detect the interaction by co-Ip of endogenous proteins using an anti-Myc antibody (Figure 3B), whereas Ip of PCNA with two different antibodies allowed more efficient detection. Differences in epitope accessibility between anti-PCNA and anti-Myc antibodies are another likely explanation for these results.

The function of c-Myc in the S phase has been attributed to the control of the initiation of DNA replication, and its overexpression induces an increase in the replication origin activity, thereby promoting DNA damage and replication stress [11]. Although PCNA is not involved in the pre-replicative complex, it is loaded at DNA replication sites during the elongation phase [12,13]. However, chromatin-bound PCNA participates in the early steps of protein degradation via the CRL4 E3 ubiquitin ligase, functioning as a docking platform for CRL4 substrates [24,25,26]. For example, PCNA interacts with proteins such as the licensing factor Cdt1, which is required for the loading of the MCM2–7 complex, and it is degraded via the CRL4 pathway in the S phase [25,27]. Given that c-Myc is also involved in events occurring before the start of DNA synthesis [11], it is conceivable that, similar to Cdt1, the interaction with PCNA may serve for degradation.

**Figure 7:**
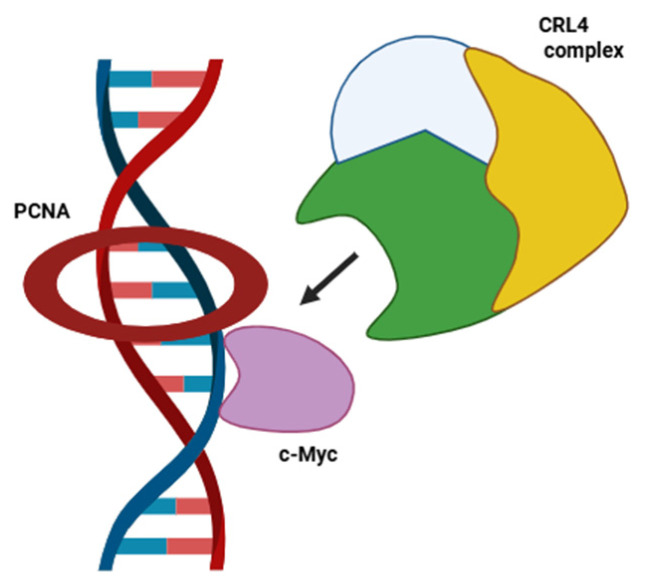
Schematic representation of the working model in which chromatin-bound PCNA (in S phase) drives c-Myc recognition by the CRL4 ubiquitin ligase complex. Created in BioRender. Maraventano, G. (2026) https://BioRender.com/f6tg5oq (accessed on 4 March 2026).

In our study, a c-Myc mutant harboring a substitution of key amino acids in the PIP box has shown increased stability compared to the wt protein. These results support a role for PCNA in driving c-Myc to the CRL4 complex for ubiquitination and subsequent degradation. Although other E3 ubiquitin ligases have been implicated in c-Myc degradation [31,32], a complex containing DDB1-CUL4A has previously been shown to regulate c-Myc stability via the substrate receptor TRUSS [33]. These observations have been recently confirmed by the identification of CUL4A in the interactome profiling of Myc protein [30]. Here, we have identified PCNA as another cofactor receptor [34], suggesting a specific role for this protein in the recognition of c-Myc substrate by the CRL4 complex during S phase. A schematic model illustrating this function is depicted in Figure 7.

Previous studies have also reported the involvement of various E3 ligases in c-Myc degradation following DNA damage [35,36]. It must be noted that the involvement of DDB1 and CUL4A in c-Myc degradation after UV damage was specifically excluded, yet no other mechanisms could be identified [37]. The discrepancy with our results may reflect differences in the cell type investigated and in the protein downregulated, i.e., DDB1 [37], vs. CUL4A in our study.

The requirement for PCNA in the recognition step of proteins that are ubiquitinated by the CRL4 complex for the proteasomal degradation was described more than a decade ago [34]. However, relatively few proteins have been identified that rely on the interaction with PCNA for their proteolysis via the CRL4 pathway [25,27]. Interestingly, this pathway operates during S phase and in all phases of the cell cycle after DNA damage [25,27]. Our study suggests that c-Myc protein is an additional substrate for the CRL4 complex using a PCNA-dependent recognition, with a mechanism similar to that described for Cdt1 [25,26,27].

Being c-Myc, an important oncogenic protein when overexpressed, its degradation during early S phase may be functional to limit the residence time at DNA replication origins. Such temporal restriction may be necessary to avoid c-Myc overfunction, which eventually leads to replication stress [7,8,11]. Several lines of evidence indicate that PCNA is required to remove DNA replication/repair proteins from chromatin [23], thereby limiting their residence on DNA during S phase or following DNA damage. Relevant examples include DNA ligase 1 [38], DDB2, XPG, as well as p12 POLD4 [39,40,41]. Notably, deregulation of this mechanism is a known source of genome instability [27,42].

In conclusion, our study identifies an additional mechanism preventing the accumulation of c-Myc. Dysfunction of this pathway may prolong and sustain c-Myc stability, thus promoting its oncogenic activity.

## 4. Materials and Methods

### 4.1. Cell Cultures and Treatments

HeLa cells (ATCC, Manassas, VA, USA) were grown in Dulbecco’s modified Eagle’s medium (DMEM) supplemented with 10% FBS (Fetal Bovine Serum) (Gibco BRL, ThermoFisher Scientific, Segrate, Italy), 100 U/100 μg streptomycin/penicillin (Gibco BRL), and 2 mM L-glutamine at 37 °C, in a 5% CO_2_ atmosphere. The human immortalized keratinocyte cell line HaCaT (cat. BS CL 168) was obtained from IZLER (Brescia, Italy) and grown in DMEM high-glucose medium supplemented with 10% FBS, streptomycin (200 μg/mL)/penicillin (200 IU), and 2 mM glutamine. Human epithelial kidney 293 (HEK 293) cells were obtained from IZLER (cat. BC CL 129) and grown in high-glucose DMEM, as above.

Exogenous expression of c-Myc was obtained using the pCMV6 expression vector, purchased from Origene (Rockville, MD, USA; cat. no. MR227353), which harbors C-terminal Myc and Flag tags. HeLa or HEK293 cells were transfected with the plasmid using Effectene reagent (Qiagen, Milano, Italy), according to the manufacturer’s protocol. In some experiments, HeLa cells were transfected with the mRFP-PCNAL2 construct, as previously described [43].

In some experiments, cells were treated with 100 μg/mL cycloheximide (CHX) purchased from Serva (Heidelberg, Germany), in the presence or in the absence of 50 μM MG132 (Sigma-Aldrich/Merck, Darmstadt, Germany), for the indicated periods of time. In DNA damage experiments, cells were exposed to UV-C irradiation (40 J/m^2^) with a TUV-9 lamp (Philips, Milano, Italy) or treated with 2.0 mM hydroxyurea (HU, Sigma-Aldrich/Merck) and collected at the indicated periods of time.

### 4.2. Site-Directed Mutagenesis

The mutation of the PIP box in the c-Myc plasmid (PCMV6) was obtained using the QuikChange II XL Site-Directed Mutagenesis Kit (Agilent Technologies, Milano, Italy), following the manufacturer’s protocol. Forward and reverse primers were, respectively, 5′-CATCATCATCGCGGACTGTGCGTGGAGCGGTGCCTCAGCCGCTG-3′ and 5′-CAGCGGCTGAGGCACCGCTCCACGCACAGTCCGCGATGATGATG-3′. Sequences were verified by automated DNA sequencing. The resulting plasmid was named c-Myc^PIPm^-Flag.

### 4.3. RNA Interference

Small-interfering (si) RNA oligos for CUL4A (ON-TARGET PLUS smart pool, cat. n. L-012610) were obtained from Dharmacon (Horizon Discovery, Cambridge, UK). HEK 293 cells were incubated for 48 h with oligos (40 nM) using INTERFERin (Polyplus, Illkirch, France) as a transfection reagent. Nontargeting siRNA (Dharmacon, cat. n. D-001810) was used as a control.

### 4.4. Western Blot, Immunoprecipitation, and Pull-Down Assay

Cells were lysed in a hypotonic buffer containing 10 mM Tris-HCl (pH 8.0), 2.5 mM MgCl_2_, 0.5% Igepal, 1 mM PMSF, and protease inhibitor cocktails (Serva) for 8 min on ice. Soluble proteins were taken apart, and nuclear pellets were incubated for 15 min at r.t with 20 U/10^6^ cells of DNase I (Sigma-Aldrich/Merck) in half the volume of initial lysis, in 10 mM Tris- HCl buffer (pH 8.0), 2.5 mM MgCl_2_, and 20 mM NaCl, as described [18]. At the end of the reaction, each fraction, or, when necessary, the total volume, was mixed with 3× loading buffer. Samples were run on nUView 8–16% gels (NuSep, Germantown, MD, USA) or Mini-Protean TGX 4–15% gels (BioRad, Hercules, CA, USA) and transferred to nitrocellulose for Western blot analysis. Membranes were blocked in 5% bovine serum albumin (BSA) in phosphate buffered saline (PBS) containing 0.2% Tween 20, then incubated for 1 h in primary antibody (listed in Supplementary Table S1) solution in blocking buffer. After three washes in the same buffer and incubation with relevant HRP-conjugated secondary antibodies (Jackson ImmunoResearch, West Grove, PA, USA). To avoid or minimize detection of heavy or light chains of Ip antibodies, Trueblot anti-mouse or anti-rabbit HRP-conjugated secondary antibodies (Rockland, Limerick, PA, USA) were also used. The membranes were incubated with chemiluminescence substrates (Cyanagen, Bologna, Italy), and images were acquired with a Westar R Imager (HiTech Cyanagen, Bologna, Italy).

For immunoprecipitation, cell extracts (0.5–1 mg protein) were either incubated with 2.5 μg anti-PCNA PC10 monoclonal antibody plus 4 μL Ab5 rabbit polyclonal antibody, or with 5 μg/mL anti-c-Myc Y69 rabbit monoclonal, or with 3 μg/mL anti-Flag M2 monoclonal antibody. For control, irrelevant mouse IgG (Sigma-Aldrich/Merck) was used at the same concentration. All antibody information is given in Table S1. Each antibody was pre-bound to protein G-beads (Dynabeads, ThermoFisher) before incubation with cell extracts. To perform immunoprecipitation of PCNA-RFP, anti-RFP Nanobody/VHH coupled to magnetic beads (RFP trap, Chromotek, Planegg, Germany) was used. The incubation with the respective extracts was performed for 3 h at 4 °C, under continuous rotation. After incubation, the immunocomplexes formed were washed three times in 50 mM Tris-HCl buffer (pH 8.0) containing 150 mM NaCl, 0.5% Igepal, 1 mM PMSF, and protease inhibitor cocktail.

Pull-down with recombinant proteins was performed using His-tagged c-Myc (Origene, cat. TP760019) and PCNA [18]. Ni-NTA beads (Qiagen) were incubated with 0.5–1 μg His-Myc, followed by a pre-clearing step in 50 mM NaH_2_P_4_ buffer (pH 8.0) containing 150 mM NaCl. Incubation of beads with bound His-c-Myc and 50 ng recombinant PCNA, or with a HeLa cell extract, was performed for 3 h at 4 °C, followed by three washings in the same buffer containing 300 mM NaCl and 20 mM imidazole. Pull-down samples were then resuspended in 60 mL of loading buffer and heated for 5 min at 90 °C.

### 4.5. Immunofluorescence and Proximity Ligation Assay

For immunofluorescence detection of c-Myc and PCNA proteins, HeLa cells seeded on coverslips were lysed in situ with hypotonic buffer containing 10 mM Tris-HCl (pH 8.0), 2.5 mM MgCl_2_, 10 mM Na β-glycerophosphate, 0.1% Igepal, 0.2 mM PMSF, and protease inhibitors for 8 min at 4 °C [18]. Cells were washed in lysis buffer without detergent and then fixed for 5 min at room temperature (r.t.) with 2% formaldehyde in PBS, followed by permeabilization in 70% ethanol at −20 °C. After blocking with 1% BSA-0.2% Tween 20 in PBS, cells were incubated for 1 h at r.t. with anti-c-Myc Y69 rabbit monoclonal antibody diluted 1:100 and anti-PCNA PC10 mouse monoclonal antibody diluted 1:200 in blocking solution. Secondary anti-mouse and anti-rabbit antibodies labeled with DyLight 594 (red fluorescence) or DyLight 488 (green fluorescence), respectively, were used (Table S1). DNA was stained with Hoechst 33,258 dye (0.1 μg/mL) in PBS, and coverslips were mounted with Mowiol. Samples were observed with a BX51 Olympus fluorescence microscope (Olympus Italia, Segrate, Italy) using a 100× oil immersion objective, and pictures were captured with an Olympus C4040 digital camera.

For the Proximity Ligation Assay (PLA), the Duolink^®^ PLA kit (Sigma-Aldrich/Merck) was used following the manufacturer’s instructions. Briefly, HeLa cells seeded on coverslips were lysed in situ, fixed with formaldehyde (2%), followed by 70% ethanol permeabilization, as described above. The coverslips were incubated in a humidity chamber (1 h at 37 °C) with the Duolink^®^ blocking solution and then with 50 μL of Duolink^®^ antibody solution containing the two primary antibodies anti-c-Myc Y69 and anti-PCNA PC10, diluted 1:100 and 1:200, respectively. For specificity control, the samples were incubated only with the PC10 antibody. In PLA on samples with exogenous Flag-tagged c-Myc WT and PIPm proteins, antibodies anti-Flag (mouse) and anti-PCNA (rabbit) were used diluted 1:200 and 1:50, respectively. After incubation with primary antibodies, coverslips were washed twice and incubated in a preheated humidity chamber for 1 h at 37 °C with PLUS and MINUS PLA probes diluted in Duolink^®^ antibody diluent. After two further washes (5 min each), samples were incubated in a preheated humidity chamber for 30 min at 37 °C with ligase in the Duolink^®^ ligation buffer for the ligation step. After washing as above, the amplification step was performed in a preheated humidity chamber for 100 min at 37 °C with polymerase diluted in the Duolink^®^ amplification buffer containing red-fluorescence-labeled nucleotides for the rolling-circle amplification. Coverslips were then washed twice, DNA was stained using Hoechst 33,258 dye, and coverslips were mounted within Mowiol, as described above. Immunofluorescence and PLA images were acquired using a confocal linear laser-scanning microscope Zeiss LSM 800 (Zeiss, Milano, Italy), using a 63× objective.

### 4.6. Statistical Analysis

At least three biological replicates (unless otherwise stated) were performed for each experiment. Statistical analysis was performed with Prism 6 software (GraphPad, San Diego, CA, USA) used to calculate significance with the Student *t* test (two-tailed), with *p* values < 0.05 considered to be significant.

## Data Availability

Data presented in this study are available upon reasonable request to the corresponding author.

## Supplementary Materials

The following supporting information can be downloaded at https://www.mdpi.com/article/10.3390/ijms27062745/s1.

## Abbreviations

The following abbreviations are used in this manuscript:

BSA	Bovine Serum Albumin
CHX	Cycloheximide
CRL	Cullin Ring E3 ubiquitin ligase
PCNA	Proliferating Cell Nuclear Antigen
PLA	Proximity Ligation Assay
PBS	Phosphate-buffered Saline

## References

[1] Grandori C., Cowley S.M., James L.P., Eiseman R.N. (2000). The Myc/Max/Mad network and the transcriptional control of cell behavior. Annu. Rev. Cell Dev. Biol..

[2] Kalkat M., De Melo J., Hickman K.A., Lourenco C., Redel C., Resetca D., Tamachi A., Tu W.B., Penn L.Z. (2017). MYC Deregulation in Primary Human Cancers. Genes.

[3] Das S.K., Lewis B.A., Levens D. (2023). MYC: A complex problem. Trends Cell Biol..

[4] Donati G., Amati B. (2022). MYC and therapy resistance in cancer: Risks and opportunities. Mol. Oncol..

[5] Weber L.I., Hartl M. (2023). Strategies to target the cancer driver MYC in tumor cells. Front. Oncol..

[6] García-Gutiérrez L., Delgado M.D., León J. (2019). MYC Oncogene Contributions to Release of Cell Cycle Brakes. Genes.

[7] Dominguez-Sola D., Ying C.Y., Grandori C., Ruggiero L., Chen B., Li M., Galloway D.A., Gu W., Gautier J., Dalla-Favera R. (2007). Non-transcriptional control of DNA replication by c-Myc. Nature.

[8] Dominguez-Sola D., Gautier J. (2014). MYC and the control of DNA replication. Cold Spring Harb. Perspect. Med..

[9] Faraco C.C.F., Zhu W., Fortier A.M., Ramdzan Z.M., Vickridge E., Ong V., Goudreau F., Djerir B., Bellemare J., Gu S. (2025). The function of MYC in base excision repair protects against RAS-induced senescence. Nucleic Acids Res..

[10] Sankar N., Kadeppagari R.K., Thimmapaya B. (2009). c-Myc-induced aberrant DNA synthesis and activation of DNA damage response in p300 knockdown cells. J. Biol. Chem..

[11] Srinivasan S.V., Dominguez-Sola D., Wang L.C., Hyrien O., Gautier J. (2013). Cdc45 is a critical effector of myc-dependent DNA replication stress. Cell Rep..

[12] Moldovan G.L., Pfander B., Jentsch S. (2007). PCNA, the maestro of the replication fork. Cell.

[13] Choe K.N., Moldovan G.L. (2017). Forging Ahead through Darkness: PCNA, Still the Principal Conductor at the Replication Fork. Mol. Cell.

[14] Witko-Sarsat V., Mocek J., Bouayad D., Tamassia N., Ribeil J.A., Candalh C., Davezac N., Reuter N., Mouthon L., Hermine O. (2010). Proliferating cell nuclear antigen acts as a cytoplasmic platform controlling human neutrophil survival. J. Exp. Med..

[15] Naryzhny S.N., Lee H. (2010). Proliferating cell nuclear antigen in the cytoplasm interacts with components of glycolysis and cancer. FEBS Lett..

[16] Bravo R., Macdonald-Bravo H. (1987). Existence of two populations of cyclin/proliferating cell nuclear antigen during the cell cycle: Association with DNA replication sites. J. Cell Biol..

[17] Leonhardt H., Rahn H.P., Weinzierl P., Sporbert A., Cremer T., Zink D., Cardoso M.C. (2000). Dynamics of DNA replication factories in living cells. J. Cell Biol..

[18] Scovassi A.I., Prosperi E. (2006). Analysis of proliferating cell nuclear antigen (PCNA) associated with DNA. Methods Mol. Biol..

[19] Mailand N., Gibbs-Seymour I., Bekker-Jensen S. (2013). Regulation of PCNA-protein interactions for genome stability. Nat. Rev. Mol. Cell Biol..

[20] Warbrick E. (1998). PCNA binding through a conserved motif. Bioessays.

[21] Dutto I., Tillhon M., Cazzalini O., Stivala L.A., Prosperi E. (2015). Biology of the cell cycle inhibitor p21CDKN1A: Molecular mechanisms and relevance in chemical toxicology. Arch. Toxicol..

[22] Georgakilas A.G., Martin O.A., Bonner W.M. (2017). p21: A Two-Faced Genome Guardian. Trends Mol. Med..

[23] Ticli G., Cazzalini O., Stivala L.A., Prosperi E. (2022). Revisiting the Function of p21(CDKN1A) in DNA Repair: The Influence of Protein Interactions and Stability. Int. J. Mol. Sci..

[24] Havens C.G., Walter J.C. (2009). Docking of a specialized PIP box onto chromatin- bound PCNA creates a degron for the ubiquitin ligase CRL4^Cdt2^. Mol. Cell.

[25] Havens C.G., Walter J.C. (2011). Mechanism of CRL4(Cdt2), a PCNA-dependent E3 ubiquitin ligase. Genes Dev..

[26] Havens C.G., Shobnam N., Guarino E., Centore R.C., Zou L., Kearsey S.E., Walter J.C. (2012). Direct Role for Proliferating Cell Nuclear Antigen in Substrate Recognition by the E3 Ubiquitin Ligase CRL4^Cdt2^. J. Biol. Chem..

[27] Mazian M.A., Yamanishi K., Rahman M.Z.A., Ganasen M., Nishitani H. (2022). CRL4(Cdt2) Ubiquitin Ligase, A Genome Caretaker Controlled by Cdt2 Binding to PCNA and DNA. Genes.

[28] Agrawal P., Yu K., Salomon A.R., Sedivy J.M. (2010). Proteomic profiling of Myc-associated proteins. Cell Cycle.

[29] Dingar D., Kalkat M., Chan P.K., Srikumar T., Bailey S.D., Tu W.B., Coyaud E., Ponzielli R., Kolyar M., Jurisica I. (2015). BioID identifies novel c-MYC interacting partners in cultured cells and xenograft tumors. J. Proteom..

[30] Kalkat M., Resetca D., Lourenco C., Chan P.K., Wei Y., Shiah Y.J., Vitkin N., Tong Y., Sunnerhagen M., Done S.J. (2018). MYC Protein Interactome Profiling Reveals Functionally Distinct Regions that Cooperate to Drive Tumorigenesis. Mol. Cell.

[31] Thomas L.R., Tansey W.P. (2011). Proteolytic control of the oncoprotein transcription factor Myc. Adv. Cancer Res..

[32] Farrell A.S., Sears R.C. (2014). MYC degradation. Cold Spring Harb. Perspect. Med..

[33] Choi S.H., Wright J.B., Gerber S.A., Cole M.D. (2010). Myc protein is stabilized by suppression of a novel E3 ligase complex in cancer cells. Genes Dev..

[34] Arias E.E., Walter J.C. (2006). PCNA functions as a molecular platform to trigger Cdt1 destruction and prevent re-replication. Nature Cell Biol..

[35] Popov N., Herold S., Llamazares M., Schülein C., Eilers M. (2007). Fbw7 and Usp28 regulate myc protein stability in response to DNA damage. Cell Cycle.

[36] Jiang M.R., Li Y.C., Yang Y., Wu J.R. (2003). c-Myc degradation induced by DNA damage results in apoptosis of CHO cells. Oncogene.

[37] Britton S., Salles B., Calsou P. (2008). c-MYC protein is degraded in response to UV irradiation. Cell Cycle.

[38] Peng Z., Liao Z., Matsumoto Y., Yang A., Tomkinson A.E. (2016). Human DNA Ligase I Interacts with and Is Targeted for Degradation by the DCAF7 Specificity Factor of the Cul4-DDB1 Ubiquitin Ligase Complex. J. Biol. Chem..

[39] Cazzalini O., Perucca P., Mocchi R., Sommatis S., Prosperi E., Stivala L.A. (2014). DDB2 association with PCNA is required for its degradation after UV-induced DNA damage. Cell Cycle.

[40] Han C., Wani G., Zhao R., Qian J., Sharma N., He J., Zhu Q., Wang Q.-E., Wani A.A. (2015). Cdt2-mediated XPG degradation promotes gap-filling DNA synthesis in nucleotide excision repair. Cell Cycle.

[41] Zhang S., Zhou Y., Trusa S., Meng X., Lee E.Y.C., Lee M.Y.W.T. (2007). A novel DNA damage response: Rapid degradation of the p12 subunit of dna polymerase delta. J. Biol. Chem..

[42] Coleman K.E., Grant G.D., Haggerty R.A., Brantley K., Shibata E., Workman B.D., Dutta A., Varma D., Purvis J.E., Cook J.G. (2015). Sequential replication-coupled destruction at G1/S ensures genome stability. Genes Dev..

[43] Cazzalini O., Sommatis S., Tillhon M., Dutto I., Bachi A., Rapp A., Nardo T., Scovassi A.I., Necchi D., Cardoso M.C. (2014). CBP and p300 acetylate PCNA to link its degradation with nucleotide excision repair synthesis. Nucleic Acids Res..

