# Supplementary material for: Stability of c-Myc Protein in Early S Phase Is Regulated by the Interaction with PCNA
Source: Int J Mol Sci. 2026 Mar 18;27(6):2745. doi: 10.3390/ijms27062745 (PMC13026809; doi:10.3390/ijms27062745)
Supplement: Supplementary file 1 [file ijms-27-02745-s001.zip › ijms-4090162-supplementary.pdf]

Supplementary File

Miriana Cardano et al.

Stability of c-Myc protein in early S phase is regulated by the interaction with PCNA.

Figure S1: Localization and electrophoretic mobility of the mutant c-Myc protein c-Myc^{PIPm}.

(A) Immunofluorescence determination of nuclear localization of exogenously expressed c-Myc^{WT}-Flag, and c-MycPIPm-Flag proteins, using anti-Flag antibody (green fluorescence). DNA is stained with Hoechst 33258 dye. Scale bar= 10 μm.
(B) Western blot analysis of electrophoretic mobility of c-Myc^{WT}-Flag, and c-Myc^{PIPm}-Flag proteins. The arrows indicates the expected position of the exogenous protein.

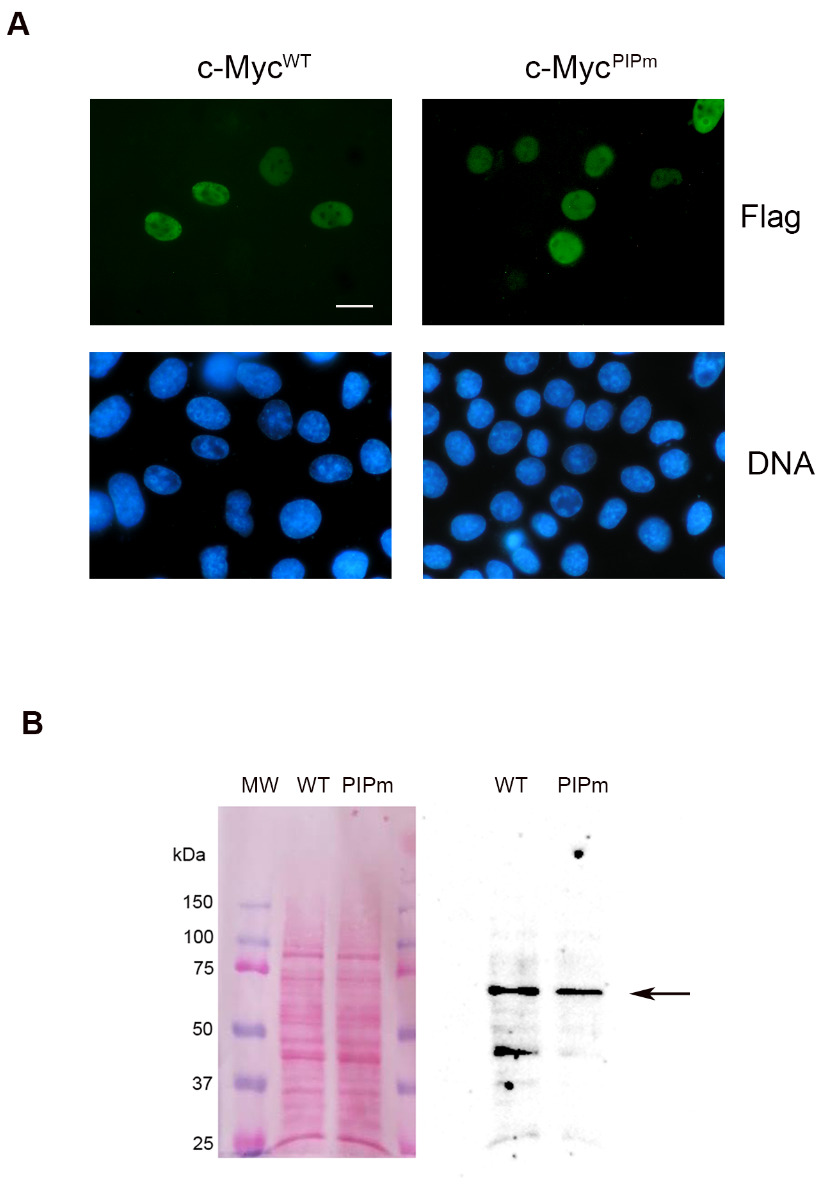

Table S1.
List of primary antibodies used in this study.

**Antigen**	**Clone**	**Company**	**Cat. No.**	**Dilution**
c-Myc	Y69	AbCam	Ab32072	1:1000
PCNA	PC10	Dako	M0879	1:1000
PCNA	Ab-5, polyclonal	Calbiochem/Merck	PC474	1:250
PCNA	polyclonal	Immunological Sci.	AB-81598	1:50
Flag	M2	Sigma/Aldrich	F1804	1:200, 1:1000
RFP	polyclonal	Rockland	600-401-379	1:2000
CUL4A	polyclonal	Genetex	GTX33129	1:500
DDB1	JU32-35	Novus Biologicals	NBP2-75465	1:1000
actin	AC40	Sigma/Aldrich	A3853	1:1000
--	IgG mouse	Sigma/Aldrich	I5381	1:500

List of secondary antibodies used in this study.

**Antibody**	**host**	**Company**	**Cat. No.**	**Dilution**
Anti-mouse HRP	goat	Jackson Immun.	115-035-146	1:5000
Anti-rabbit HRP	goat	Jackson Immun.	111-035-144	1:10000
Anti-mouse HRP Trueblot	rat	Rockland	18-8817-33	1:1000
Anti-rabbit HRP Trueblot	mouse	Rockland	18-8816-31	1:1000
Anti-mouse DyLight 594	goat	Thermo Fisher	35510	1:100
Anti-rabbit DyLight 488	goat	Thermo Fisher	35552	1:100
